# Developing a communication curriculum for primary and consulting services

**DOI:** 10.1080/10872981.2020.1794341

**Published:** 2020-07-21

**Authors:** Michelle A Lopez, Judith Campbell

**Affiliations:** Department of Pediatrics, Baylor College of Medicine, Houston, Texas, USA

**Keywords:** Medical education, consultant, inter-subspecialty, curriculum, communication

## Abstract

Communication skills are fundamental to effective patient care, and inter-subspecialty communication is frequently identified as a key component of medical education curriculums globally. The team primarily responsible for a patient, the ‘primary service’, may often request a consult from a specialist, the ‘consulting service’, for questions of diagnosis, management, or assistance in arranging or performing a procedure or test. Few resources exist to support the development and growth of communication curriculums across primary and consulting services. We provide tips to improve communication across services in patient care and enhance learning for multiple levels of providers. This article provides a guide for the planning and implementation of a communication curriculum and highlights key components for success, based on our experience as teaching faculty on primary and consulting services, at a large academic institution. With the proper collaborations, teaching touch points, specialist consult communication tool, peer coaches, and timely feedback, this course can meet numerous educational and institutional priorities.

## Introduction

Communication skills are fundamental to providing effective patient care, and within communication, providers must be able to seek the guidance and expertise of other subspecialists when needed through a consult. The team primarily responsible for a patient, the “primary service”, may often request a consult from a specialist, the “consulting service”, for questions of diagnosis, management, or assistance in arranging or performing a procedure or test [1]. As identified in the 1957 American Medical Association Principles of Medical Ethics, ‘A physician should seek consultation upon request; in doubtful or difficult cases; or whenever it appears that the quality of medical service may be enhanced thereby’ [2]. Collaboration amongst colleagues is consistently identified as a key concept in medical training by international accrediting bodies such as the Accreditation Council for Graduate Medical Education (ACGME) [3], World Federation of Medical Education [4], the Royal College of Physicians and Surgeons of Canada [5], and the UK General Medical Council Competencies [6]. Both the Association of Pediatric Program Directors (APPD) and the American Board of Pediatrics (ABP) have identified consultation and referral to other subspecialists when needed as an essential entrustable professional activity (EPA) for graduating pediatric residents [7]. Trainees are the frontline of patient care in academic institutions, and educational efforts targeting trainees are the first priority in the process of improving communication amongst primary and consulting services. Existing medical education literature has examined the key elements of the requesting a consult from a specialist and created tools to aid trainees in calling consulting services [8-10]. However, literature providing guidance on how to incorporate education on communicating with consulting services, particularly beyond medical students, is lacking. Developing skills to enhance communication across the primary and consulting services is crucial in undergraduate medical education (UME) and at the graduate medical education (GME) levels. Furthermore, teaching faculty are crucial figures in role-modeling behavior when calling and receiving specialist consults, providing real-time feedback, and improving practice that requires an engagement of all members of the larger team.

**Figure 1.**
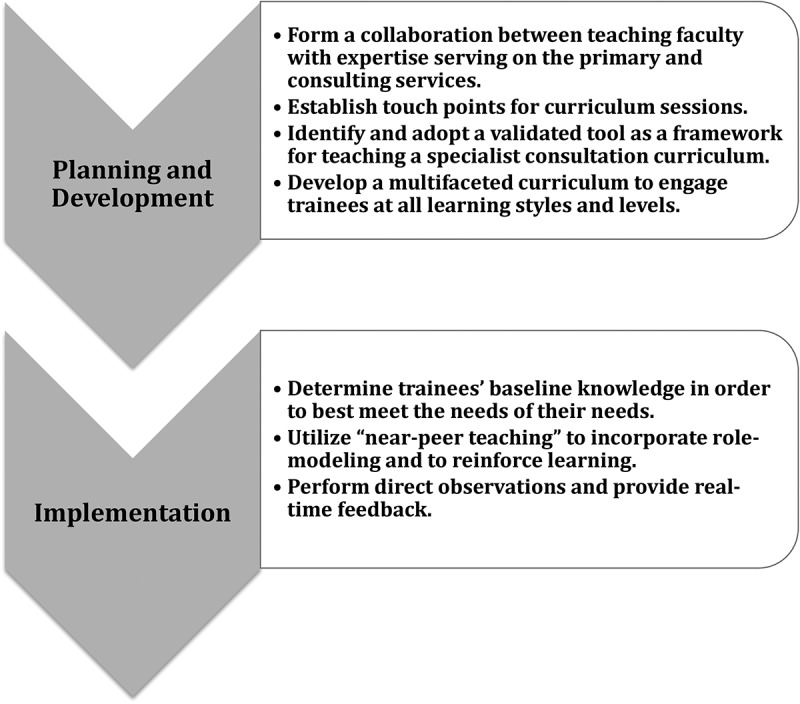
Guide to planning, developing, and implementing a communication curriculum for primary and consulting services.

In response to this need, we present a guide aimed at numerous levels of medical providers. Review of communication fundamentals, consideration of challenges in the role of the primary and consulting services, and implementation of communication tools enable all members of the team to develop a shared mental model of their roles and responsibilities in the specialist consult communication process. We provide tips for planning and implementation [Figure 1] based on our experiences and existing literature with the goal of enhancing communication across primary and consulting services and improving learning for several levels of providers (UME, GME, and teaching faculty).

## Planning and development

### Form a collaboration between educators with expertise serving on the primary and consulting services

A successful educational curriculum for primary and consulting services first involves creating and modeling effective collaborations. Collaboration requires interdependency, rather than autonomy, and a sharing of knowledge and skills [11]. Teaching faculty have individual understandings of the challenges that they face serving as the primary or consulting service. At our institution, we noted a theme on teaching evaluations in UME which highlighted the negative views that some services often shared about each other in front of trainees. This led to several corrective responses. One of the most powerful demonstrations of the importance of respecting each other’s roles and contributions was forming a collaboration between the Pediatric Hospital Medicine and Infectious Disease teaching faculty. This allowed us to model collegiality and an appreciation of each other’s perspectives. The unique experiences that various faculty members bring to the educational curriculum provide balance and model the strengths of effective collaboration across specialties. *Collaboration is the foundation for a curriculum and requires engagement of both primary and consulting services*.

### Establish touch points for curriculum sessions

In the planning stage, it is important to consider ideal curriculum touch points, and specifically, the key times for instruction within the overall training period and within the academic year. Recent literature using a specialist consulting service curriculum within UME suggests a staged curriculum across first year (pre-clinical), second year (core clinical), and fourth year (pre-intern year) of medical school [12]. We found it crucial to condense this information and implemented the curriculum at the GME level, in the first year of training, in three interactive sessions. At the first session, we provided an introduction to the skill of communicating with specialists on consulting services. At the second session, trainees learned key components of requesting a specialist consult, received a laminated card with these components, practiced communication skills, and completed a reflective exercise based on past experiences with consulting services. At the third session, trainees reviewed cases in which poor communication with consulting services negatively impacted patient safety; trainees identified opportunities for improvement. Based on feedback from the trainees about the challenges of learning and practicing this curriculum on a busy clinical service, we introduced key didactics during protected time such as orientation or educational retreats. *We recommend evaluating the needs of the local trainees and implementing the curriculum in settings and timeframes that best suit their needs.*

### Identify and adopt a validated tool as a framework for teaching a specialist consultation curriculum

A tool that identifies the key elements of calling a specialist consult provides structure to an educational curriculum for working with consulting services and supplies a useful reference for trainees. Additionally, this tool provides an objective measure for delivering feedback to trainees on their implementation of the required skills. A previous survey of physicians found that providers agreed on several of the essential components for effective specialist consults [13], and several models have emerged as tools for requesting assistance from a consulting service. For example, Kessler et al. developed the five Cs of a Consultation Model–Contact, Communicate, Core Question, Collaboration, and Close the Loop–based on a theoretical framework, the 7Cs of a Consultation Business Model, existing evidence-based literature, and expert opinion [14]. The PIQUED model by Chan et al. focuses on Preparation, Identification, Questions, Urgency, Educational Modifications, and Debrief/Discuss [15]. This model was created from focus group interviews and designed to increase the educational value of the consult [15]. Kessler, Chan, and others also created a COMBINED model of the essential elements in 5Cs and PIQUED [8]. An additional model for consideration is CONSULT by Podolsky et al., which involves Contact, Orient, Narrow Question, Story, Urgency, Later, and Thank you! [10]. This model was based on feedback from trainees on their experiences calling consulting services [10]. As specialist consult curriculums continue to be recognized as a priority in medical training, additional tools likely will emerge. *We recommend that educators review the existing tools with local stakeholders and determine the best fit for their institution, as having an effective tool is a critical component in building a curriculum to teach the art of requesting specialist consults.*

### Develop a multifaceted curriculum to engage trainees at all learning styles and levels

We created a multifaceted experience involving didactics, skill-sets practice, a learning prompt, role play to explore the perspectives of different members of the team, and reflective exercises. The multimodal teaching techniques were incorporated to engage trainees who respond best to various teaching styles. Similarly, a pilot training program in a pediatric GME program successfully conducted workshops on referral and communication skills [16]. It included practicing a case requiring subspecialty referral [16]. The workshop ended with evaluation and feedback sessions and found an improvement in trainees’ confidence and communication skills in simulation [16]. In our experience, we found the reflective exercises to be particularly valuable. Across first-year GME trainees, 30% reported ‘rude’ responses from consulting services, 60% identified challenges in the process and/or practice of communicating with consulting services, 50% noted they did take time to teach, and 60% appreciated clear verbal communication of recommendations. This feedback was used to guide the content development for the GME and teaching faculty workshops. Overall, our trainees rated the communication skills workshop as likely to enhance their care of patients. *Multifaceted training experiences are important components of the specialist consult communication curriculum*.

## Implementation

### Determine trainees’ baseline knowledge in order to best meet their needs

**Figure 2.**
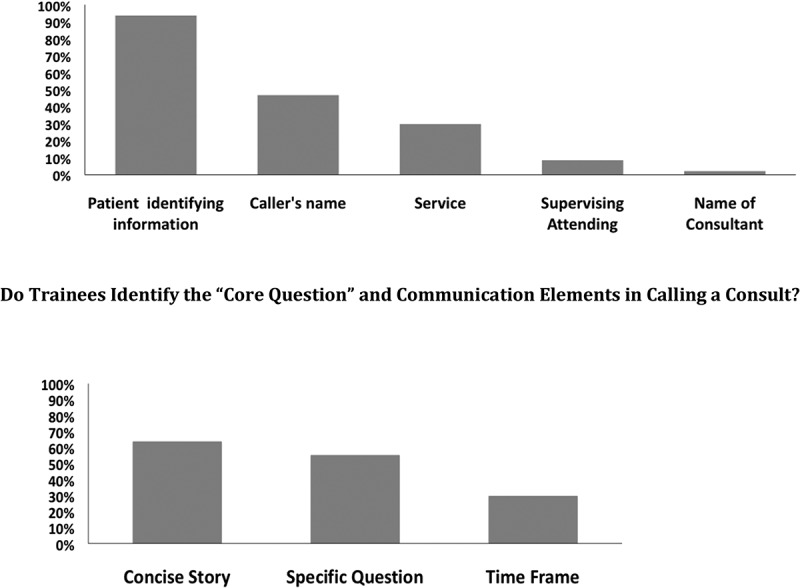
Needs assessment with first-year graduate medical education trainees.

We started teaching sessions with an assessment of the trainees’ baseline knowledge. Testing first years in GME on their knowledge of the key components identified in Kessler’s 5C’s [9], we found that they rarely self-identified the importance of obtaining the name and callback information for the consulting service when considering the ‘contact’ elements (Figure 2). In an examination of the communication elements, only 55% of trainees reported the need for a core question and most trainees lacked understanding of the importance of a consult time frame (Figure 2). We were able to tailor portions of the teaching session to focus on these identified deficits. *We recommend starting the teaching session with an assessment of baseline knowledge*.

Do Trainees Identify the Essential ‘Contact’ Elements in Calling a Consult? Do Trainees Identify the ‘Core Question’ and Communication Elements in Calling a Consult?

### Utilize ‘near-peer teaching’ to incorporate role-modeling and to reinforce learning

Upper level GME trainees serve as ‘near-peer’ coaches for first-year GME trainees and UME trainees. A near-peer coach is a peer because they are also a trainee, but they are an upper level trainee, and thus more advanced in their own skill development. Using near-peers as teachers has numerous benefits, one of which is to allow the ‘learner-turned-teacher to learn twice’ [17], allowing the upper level GME trainee to solidify their knowledge. Near-peer teaching can be used within a primary or consulting service, and they can also be used across services. For example, upper level GME trainees, such as fellows from consulting services who teach UME and GME trainees on the primary service, have been shown to improve their own teaching skills [18]. In our setting, upper level GME trainees on the consulting services were also effective in providing real-time and focused feedback to trainees on the primary service when they called to request consulting services. Additional benefits of a near-peer teaching model include spreading teaching opportunities among several members of the team, providing education on the cognitive level of the learner, creating a safe and comfortable learning environment, enhancing intrinsic motivation, and preparing physicians for their future roles as educators [19]. Leveraging relationships between upper level and first-year GME trainees provide a less intimidating learning environment and provides benefits for the teacher and learner. *Hence, we highly recommend near-peer teaching to develop skills for requesting assistance from consulting services.*

### Perform direct observations and provide real-time feedback

Evaluation of what was learned is another key component of the learning experience and is critical for individual self-improvement [20]. A structured process for feedback is particularly warranted when implementing the use of a new tool or skill set, and includes the source, timing, and valence of the feedback [21]. The capacity of the trainee for feedback will also impact how the feedback is received. Previous work with specialist consult curriculums found a simulated environment to be an effective and safe setting for feedback in preparing first-year GME trainees to request consultations, and this was well received by the trainees [22]. Additionally, it is important to consider who is providing the feedback. A curriculum with mock paging found success with several layers of feedback including self-reflection, peer, nurse practitioner, and teaching faculty feedback followed by direction on specific next steps in improvement [23]. As the new skill is taught, trainees should be given an opportunity to practice the skills in a safe learning environment with peers or standardized patients. Feedback during the workshop using role play and self-observation/reflection is key. *As the trainees begin to incorporate the use of the skills, real-time observations and feedback can lead to quicker mastery of the skills*.

## Conclusions

Communication across subspecialties is a fundamental communication skill for trainees to master. The need to develop this skill is highlighted in the educational competencies within several medical curriculums globally and is a key component of patient care. During medical training, institutions have the opportunity to optimize trainees’ communication skills across the primary and consulting services. We provide tips on fundamental components of a specialist consult communication course based on our experiences and existing literature. Future work is needed in evaluating a robust curriculum across multiple levels of trainees.
